# Determinants Impacting Daily Physical Activity Levels Among Chinese Adults and Its Association with Obesity

**DOI:** 10.3390/healthcare13233027

**Published:** 2025-11-24

**Authors:** Yizhi Tang, Sihan Ruan, Xihan Zhou, Jiayi Chen, Xiaoxiao Wu, Qi Zhu

**Affiliations:** Department of Medicine, Xinglin College, Nantong University, Nantong 226019, China; 2002142666@stmail.ntu.edu.cn (Y.T.); ruansihan@stmail.ntu.edu.cn (S.R.); 2030141457@stmail.ntu.edu.cn (X.Z.); 2033143065@stmail.ntu.edu.cn (J.C.); 2030141793@stmail.ntu.edu.cn (X.W.)

**Keywords:** physical activity, obesity, impact factors

## Abstract

**Background/Objectives:** This study investigated the influencing factors of daily physical activity among Chinese adults and its association with obesity. **Methods:** A nationwide online questionnaires survey was conducted using Question star, involving 863 Chinese adults. One-way χ^2^ test, analysis of variance, and logistic regression were employed to explore the determinants of physical activity. Additionally, chi-square test and *t*-test were utilized to compare and analyze the impact of physical activity on obesity. **Results:** Males reported significantly higher physical activity levels than females (e.g., total score: 57.19 vs. 52.89, *p* < 0.001). Participants in the 28~37 years age group had the highest activity scores, which were significantly greater than those in older groups (38~58 years, *p* < 0.001). Both higher income (*p* = 0.018) and educational attainment (*p* = 0.001) were positively associated with physical activity engagement The non-obese population demonstrated significantly better performance in terms of daily physical activity compared to the obese population, particularly for question Q1 (*p* = 0.016), Q5 (*p* = 0.005), Q6 (*p* = 0.021), Q7 (*p* = 0.01), and Q8 (*p* = 0.03). **Conclusions:** Interventions aimed at promoting daily physical activity among adults should prioritize women, individuals with obesity, those with lower income levels, and those with limited educational attainment. This study provides a validated tool (the Daily Physical Activity Behavior Scale, DPABS) and targeted behavioral insights to support the design of feasible, daily physical activity interventions for obesity prevention in Chinese adults.

## 1. Introduction

Rising living standards in China have precipitated significant shifts in lifestyle and dietary patterns, contributing to a marked increase in the prevalence and incidence of overweight and obesity among adults [1]. This trend represents a substantial public health burden, driving associated comorbidities such as hyperlipidemia and coronary heart disease [2]. As established primary risk factors for numerous pathologies impacting joint [3], cardiovascular [4], and psychological health [5], the escalating overweight/obesity rates threaten population health and quality of life, directly impeding national “Healthy China 2030” objectives. Consequently, effective body weight management strategies are paramount. Within a social–ecological and behavioral framework such as Social Cognitive Theory, physical activity (PA) is widely recognized as a cornerstone intervention [2], influenced by a dynamic interplay of personal, environmental, and behavioral factors.

However, structured exercise regimens (e.g., gym-based training) present significant accessibility barriers for contemporary adults, particularly those facing high work/study pressures in economically dynamic regions like the Yangtze River Delta. While evidence suggests modest increases in daily moderate-to-vigorous PA (MVPA; e.g., 5–10 min) associate with reduced obesity risk [6], and fitness maintenance correlates with PA adherence [7], a critical knowledge gap persists. Specifically, the impact of discrete, quantifiable daily life-integrated PA behaviors on weight management, and their distinct relationship to obesity status within populations constrained by time, remains inadequately characterized. Furthermore, while socio-demographic determinants like income and education influence PA initiation and maintenance, particularly for women [8], their specific association with daily life-integrated PA patterns in Chinese adults requires deeper elucidation. This study, therefore, specifically investigates the following:

1. The socio-demographic determinants (gender, age, education, income, marital status, fertility status) significantly associated with daily life-integrated PA levels among Chinese adults.
2. The association between specific, quantifiable daily life-integrated PA behaviors (measured via DPABS items Q1-Q9) and obesity status (defined by BMI).
3. The specific daily PA behaviors exhibiting the most significant differences between obese and non-obese individuals.

Based on existing evidence and the identified gap, we hypothesize that:

**H1:**  *Higher daily life-integrated PA levels will associate significantly with male gender, younger–middle age (28–37 years), higher education, and higher income.*

**H2:**  *Non-obese individuals will demonstrate significantly higher engagement in specific daily life-integrated PA behaviors requiring planning, consistency, and barrier mitigation (e.g., achieving weekly aerobic targets [Q1], maintaining backup plans [Q5], compensating for missed sessions [Q6], creating exercise opportunities [Q7], prioritizing PA [Q8]) compared to obese counterparts.*

**H3:**  *The associations posited in H1 and H2 will persist after controlling for confounding socio-demographic factors.*

The primary objective of this study is to identify key socio-demographic and behavioral determinants of daily life-integrated PA, measured via the DPABS, and to rigorously examine their specific associations with obesity status (operationalized by BMI categories) among Chinese adults, focusing on feasible behaviors embedded within everyday routines.

This research holds significant value by:

1. Providing actionable evidence for developing targeted interventions promoting feasible, integrated PA strategies for weight management, particularly for time-constrained populations. This addresses a critical need in combating China’s obesity epidemic.
2. Informing public health policy by highlighting modifiable socio-demographic factors (e.g., targeting women, lower-income, lower-education groups) and effective behavioral targets for population-level PA promotion.

Offering substantial originality:

1. Focus on Daily Integration: Moving beyond structured exercise or specific sports [4], we uniquely quantify the role of integrating PA opportunities into daily routines (e.g., active transport, stair use, opportunistic movement, domestic activities) for weight management in the Chinese context.
2. Granular Behavioral Assessment: We employ a novel, context-specific instrument (Daily Physical Activity Behavior Scale—DPABS) to quantify nine distinct, common daily life-integrated PA behaviors and directly link them to obesity status, providing specificity often absent in broader PA metrics.
3. Contextual Relevance: The study explicitly targets contemporary Chinese adults, addressing challenges prevalent in high-pressure, time-scarce environments typified by the Yangtze River Delta region.

By addressing these specific hypotheses and research questions, this study aims to generate actionable insights for promoting accessible and effective physical activity as a fundamental strategy against obesity within the daily lives of Chinese adults.

## 2. Methods

### 2.1. Research Design and Investigate Objects

A survey of adults was conducted from September 2022 to November 2022 in the Yangtze River Delta region, employing a web-based questionnaire distributed via Question star. Given resource constraints and the exploratory nature of this study, we adopted a convenience sampling approach with voluntary participation. We acknowledge that this method may introduce selection bias and limit the generalizability of our findings. The respondents were selected based on criteria of voluntary participation and ensuring their comprehension of the questionnaire content and independent completion. We acknowledge that online recruitment may underrepresent populations with limited digital access; this limitation is addressed in Section 4.6. This study employs a descriptive correlational research approach within the framework of cross-sectional research design. This study has been reviewed and approved by the Ethics Committee of Nantong University. The experimental design and implementation plan have been meticulously developed to ensure safety and equity principles. The research content poses no harm or risk to participants, and recruitment will be conducted strictly based on voluntary participation and informed consent. Maximum measures will be taken to protect participant privacy, and there are no conflicts of interest in either the research content or outcomes. (Ethics Approval Number: 202271)”

### 2.2. Investigation Methods and Contents

#### 2.2.1. Social Demography Information

The survey collected comprehensive demographic and anthropometric data. Demographic information included gender, age, residence, occupation, education level, household income, marital status, fertility status, chronic disease status. Anthropometric measurements included height and weight. Although clinical measurements would strengthen validity, logistical constraints necessitated self-reported anthropometrics. Prior studies support moderate validity of self-reported BMI in Chinese adults [9]. The respondents themselves provide data for height and weight, which are subsequently utilized to compute the body mass index (BMI). BMI is calculated as the ratio of body weight (kg) to the square of height (m). According to the “People’s Republic of China Health Industry Standard for Adult Weight Determination”, individuals with a BMI ≤ 18.5 are classified as underweight, those with a BMI between 18 and 23.9 fall within the normal weight range, individuals with a BMI between 24 and 27.9 are categorized as overweight, while those with a BMI ≥ 28 are considered obese.

#### 2.2.2. Questionnaire of Daily Physical Activity

The Daily Physical Activity Behavior Scale (DPABS) was developed to assess common, feasible PA behaviors integrated into daily life, a domain not comprehensively captured by existing instruments focused on structured exercise or total energy expenditure. Item generation was based on a review of relevant literature on behavioral theory and weight management, e.g., [10,11], and refined through a structured expert review process involving five public health and behavioral science specialists. Content Validity Index (CVI) was calculated at 0.89. Pilot testing was conducted with a sample of 50 adults (age range: 25–45; 52% female; mixed educational backgrounds) to assess item clarity, relevance, and comprehensibility. Feedback from the pilot phase was used to refine the wording of several items. The final instrument consisted of nine items targeting key behavioral domains as follows: aerobic goal attainment (Q1), opportunistic activity (Q2, Q3, Q9), environmental support (Q4), and self-regulatory skills (planning: Q5; consistency: Q6; proactivity: Q7; prioritization: Q8).

The nine items are as follows:

Q1: Perform at least 150 min of moderate intensity aerobic exercise (e.g., brisk walking, jogging, etc.) per week.

Q2: Climbing stairs instead of taking the elevator in a building of 7 floors or less.

Q3: Whenever possible, walk instead of drive and/or take the car for short-distance trips.

Q4: Putting some sports equipment (e.g., small dumbbells) at home or in the office so that you can exercise at any time.

Q5: Have an alternate indoor exercise program when the weather does not allow outdoor exercise.

Q6: If I miss a day of exercise due to force majeure, I will make up for it by increasing the amount of time spent exercising on another day.

Q7: Try to create opportunities for exercise, e.g., while watching TV or playing on the cell phone.

Q8: Exercise even if I have a lot of things to do.

Q9: Use housework, shopping, etc., as a form of exercise.

Content validity was established through expert review (CVI = 0.89), and pilot testing (n = 50) confirmed item clarity.

The Daily Physical Activity Behavior Scale (DPABS) questionnaire utilized a 5-point Likert scale, with responses ranging from “never” as 1, “seldom” as 2, “sometimes” as 3, “often” as 4, and “always” as 5 for entries favorable to weight management and the opposite for unfavorable entries. The DPABS consisted of 9 items assessing physical activity time, mode, and habits. The Cronbach’s alpha coefficient of the questionnaire was calculated at 0.888. While we recognize the value of objective measures (e.g., accelerometers), their implementation was infeasible at this scale; future studies should incorporate device-based validation.

### 2.3. Statistical Analysis

The validated questionnaires in this study were analyzed using SPSS 27.0 software. Missing data (<5% for all variables) were handled using pairwise deletion, which utilizes all available data for each specific analysis but assumes data are missing completely at random (MCAR). We conducted a preliminary analysis which showed no systematic pattern in missingness.

Post hoc power analysis confirmed >80% power for main effects but limited power (58%) for obese subgroup analyses. Measurement data were presented as X ± s (Mean ± Standard Deviation), while count data were expressed as frequency and constitutive ratio. T-test with Welch’s analysis of variance was employed for analyzing measurement information, chosen for its robustness against departures of homogeneity of variance and unequal sample sizes across comparison groups (e.g., age and income categories). Chi-square test was used where multiple comparisons occurred (e.g., Table 1); we report uncorrected *p*-values but interpret findings conservatively given increased Type I error risk. Multiple linear stepwise regression analysis was applied for multifactorial analysis.

## 3. Result

### 3.1. General Information of the Survey Respondents

A total of 1113 original questionnaires were collected; exclusions (n = 250) resulted from incomplete responses (68%), inconsistent answer patterns (22%), and age < 18 years (10%). The final number of questionnaires that could be used for analysis was 863, with a questionnaire pass rate of 77.5%. Among them, 350 were male, accounting for 40.6%. There were 513 females, accounting for 59.4%. Then, 53.0% of the population aged 18–27, 16.3% of the population aged 28–37, 20.0% of the population aged 38–47, 7.4% of the population aged 48–57, and 3.2% of the population aged 58 years or older were surveyed. The small obese subgroup (n = 58) and limited older adult representation constrain subgroup analyses; findings should be interpreted as preliminary.

### 3.2. Fundamental Aspects of the Daily Physical Activity

The survey was conducted using a questionnaire, wherein the comprehensive responses and score distribution for each item were based on a 5-point Likert scale (ranging from 1 = never to 5 = always).

Q1: “Perform at least 150 min of moderate-intensity aerobic exercise (e.g., brisk walking, jogging, etc.) per week” was selected by “Never” = 8.1%, “Rarely” = 31.1%, “Sometimes” = 36.3%, “Frequently” = 17%, “Always” = 7.5%, with a score of 2.85 ± 1.04;

Q2: “Climbing stairs instead of taking the elevator in a building of 7 floors or less” Option “Never” = 12.7%, “Rarely” = 30.4%, “Sometimes” = 29.2%, “Frequently” = 17.4%, “Always” = 10.3%, with a score of 2.82 ± 1.17;

Q3: “Whenever possible, walk instead of drive and/or take the car for short-distance trips” the choice option “Never” = 4.3%, “Rarely” = 21.1%, “Sometimes” = 35.8%, “Frequently” = 27.5%, “Always” = 10.9%, with a score of 3.2 ± 1.03;

Q4: “Putting some sports equipment (e.g., small dumbbells) at home or in the office so that you can exercise at any time” Selected options “Never” = 21.3%, “Rarely” = 32.3%, “Sometimes” = 31.5%, “Frequently” = 9.8%, “Always” = 5.0%, with a score of 2.45 ± 1.08;

Q5: “Have an alternate indoor exercise program when the weather does not allow outdoor exercise”, the choice of option “Never” = 15.2%, “Rarely” = 32.2%, “Sometimes” = 35.8%, “Frequently” = 11.5%, “Always” = 5.0%, with a score of 2.59 ± 1.04;

Q6: “If I miss a day of exercise due to force majeure, I will make up for it by increasing the amount of time spent exercising on another day”, “Never” = 16.8%, “Rarely” = 37.9%, “Sometimes” = 33.6%, “Frequently” = 8.6%, “Always” = 3.1%, with a score of 2.43 ± 0.97,

Q7: “Try to create opportunities for exercise, e.g., while watching TV or playing cell phone”, “Never” = 12.1%, “Seldom” = 33%, and “Sometimes” = 38.1%, “Frequently” = 13.4%, “Always” = 3.4%, with a score of 2.63 ± 0.97;

Q8: “Exercise even if I have a lot of things to do” The percentage of choosing the option “never” = 13.9%, “seldom” = 34.8%, “sometimes” = 36%, “Frequently” = 11.5%, and “always” = 3.8%, with a score of 2.57 ± 0.99;

Q9: “Use housework, shopping, etc. as a form of exercise” selection option “Never” = 7.3%, “Rarely” = 18.7%, “Sometimes” = 43.2%, “Frequently” = 23.9%, “Always” = 7.0%, with a score of 3.05 ± 1.

### 3.3. The Results of the ANOVA for Examining the Impact of Each Socio-Demographic Factor on Daily Physical Activity

ANOVA results revealed that age and education level were significantly associated with scores across all nine DPABS items (all *p* < 0.05). Income level significantly influenced several behaviors, particularly those related to structured planning and opportunistic activity (Q1, Q3, Q4, Q5, Q7, Q8, Q9). Fertility status was primarily associated with weekly aerobic exercise (Q1) and viewing housework as exercise (Q9). Key demographic differences are summarized in Table 1.

### 3.4. The Results of the Multifactorial Analysis Investigating the Impact of Socio-Demographic Factors on Daily Physical Activity

The physical activity score was used as the dependent variable in multiple linear stepwise regression analyses, while socio-demographic factors were considered as independent variables. The inclusion level for variables was set at *p* < 0.05 and the exclusion level at *p* > 0.1. Results revealed that all the six factors included in the analysis had a significant impact on the physical activity score, explaining 11.0% of its variance. Educational attainment (1 = junior high school and below group, 2 = high schools and junior colleges, 3 = junior college, 4 = undergraduate, 5 = master and above group), personal income (1 = less than 3000 yuan/month, 2 = 3000 to 8000 yuan/month, 3 = 8000 to 15,000 yuan/month, 4 = 15,000 to 30,000 yuan/month, 5 = greater than 30,000 yuan/month), and childbearing status (1 = no children, 2 = 1 child, 3 = 2 children and above) were significantly and positively associated with physical activity scores. On the other hand, gender factor (1 = male, 2 = female), age (1 = 18–28 years old, 2 = 28–38 years old, 3 = 38–48 years old, 4 = 48–58 years old, 5 = 58~years old), and marital status (1 = married, 2 = unmarried) were significantly negatively correlated with physical activity scores. (See Table 2 for details.)

### 3.5. Relevance of Daily Physical Activity in the Context of Obesity

#### 3.5.1. Comparison of the Chi-Square Test for Daily Physical Activity Entries Between Obese and Non-Obese Groups

The results significantly indicated associations between obesity and the following questionnaire items: Q1 (*p* = 0.016), Q5 (*p* = 0.005), Q6 (*p* = 0.021), Q7 (*p* = 0.01), and Q8 (*p* = 0.003). Specifically, among these items, “Q1 Perform at least 150 min of moderate-intensity aerobic exercise per week” exhibited a significantly higher prevalence of “never” in the obese group (19%) compared to the non-obese group (7.3%). Additionally, for “Q5 Have an alternate indoor exercise program when the weather does not allow outdoor exercise”, the rate of choosing “never” was significantly higher in the obese group (31%) than in the non-obese group (14%), while the rate of selecting “frequently” was significantly higher in the non-obese group (12.4%) compared to the obese group (3.4%). Furthermore, “Q6 If I miss a day of exercise due to force majeure, I will make up for it by increasing the amount of time spent exercising on another day” showed a significantly higher endorsement in the obese group (31%) than in the non-obese group (15.8%). Moreover, for “Q7 Try to create opportunities for exercise, e.g., while watching TV or playing cell phone” individuals from the obese group chose “never” at a significantly higher rate (25.9%) than those from the non-obese group (11.1%). Finally, “Q8 Exercise even if I have a lot of things to do”, participants from the obese group selected “never” at a substantially higher rate (27.6%) than those from the non-obese group (12.9%), while the prevalence of choosing “frequently” was considerably higher in the non-obese group (12.2%) compared to the obese group (1.7%). (See Table 3 for details.)

#### 3.5.2. Comparison of Daily Physical Activity Scores Between Groups with and Without Obesity

The results indicated that the non-obese group achieved significantly higher scores than the obese group in entries Q1, Q4, Q5, Q6, Q7, and Q8 (refer to Table 4 for detailed information).

## 4. Discussion

This study provides novel insights into the socio-demographic determinants of daily life-integrated physical activity (PA) and its specific associations with obesity status among Chinese adults. By focusing on feasible, routine-integrated behaviors rather than structured exercise, we identified key groups (women, older adults, lower-income, lower-education individuals, and obese individuals) exhibiting lower PA engagement and pinpointed specific behaviors most strongly linked to obesity. Our findings have significant implications for targeted public health interventions within the context of China’s rising obesity burden and the “Healthy China 2030” initiative. Our results provide strong support for our initial hypotheses. H1 was fully supported, with PA levels being significantly higher among males, middle-young adults (28–37 years), and those with higher education and income. H2 was also confirmed, as non-obese individuals reported significantly greater engagement in PA behaviors requiring planning, consistency, and prioritization (Q1, Q5–Q8). Finally, H3 was upheld, as these associations remained significant after controlling for key socio-demographic confounders in our regression model.

### 4.1. PA–Obesity Relationship: Potential Mechanisms

The robust association we observed between lower daily life-integrated PA and higher obesity prevalence (particularly for behaviors Q1, Q5, Q6, Q7, Q8) likely operates through intertwined physiological, behavioral, and psychosocial mechanisms.

Physiological: Reduced PA directly lowers total energy expenditure, creating a positive energy balance crucial for weight gain and maintenance [12,13]. Beyond simple calorie deficit, PA enhances insulin [14] sensitivity, improves lipid profiles, promotes mitochondrial biogenesis [15].

Behavioral: The specific behaviors showing strong differences (Q1: achieving aerobic targets; Q5/Q6: planning/consistency; Q7/Q8: proactive integration/prioritization) reflect core elements of successful habit formation and self-regulation [16]. Obese individuals may struggle more with these aspects, leading to lower PA adherence. Our finding that non-obese individuals scored higher on these items suggests that possessing these behavioral skills is protective against obesity.

Psychosocial: Lower self-efficacy (confidence in overcoming barriers like bad weather or busy schedules, reflected in Q5–Q8) and perceived lack of time or social support are well-documented barriers to PA, particularly among women and lower-SES groups [17,18]. These factors can create a negative cycle where low PA contributes to weight gain, which in turn may further decrease motivation and self-efficacy due to discomfort or perceived difficulty.

### 4.2. Socio-Demographic Determinants: Explanations and Critique

Our findings align with, but also add nuance, to the existing literature:

Gender Disparity: Consistent with other studies [19], males exhibited significantly higher PA levels across most behaviors. While biological factors like muscle mass may play a role [20], this disparity is likely heavily influenced by sociocultural factors in China. Women often bear a disproportionate burden of domestic responsibilities and childcare [21], potentially limiting time and energy for PA. Furthermore, societal norms regarding femininity and physical exertion may also act as barriers. Interventions must explicitly address these gendered constraints.

Age Gradient: The peak PA in the 28–37 age group, followed by declines, particularly sharp after 58, is noteworthy. While age-related physiological decline contributes [22,23], our findings partially contrast with studies emphasizing the benefits of PA in older age [24,25,26]. The starkly lower scores in the oldest group (>58 years) suggest that in the high-pressure Yangtze Delta context studied, factors like increased chronic disease burden, reduced mobility confidence, safety concerns, or lack of age-appropriate PA opportunities may be significant barriers not fully overcome by general awareness of benefits. This highlights the need for context-specific understanding beyond broad age-PA correlations.

Education and Income: The positive association between higher education/income and PA engagement confirms findings like Alley, S.J et al. [27] and aligns with the Resource Model, e.g., Dong, J et al. [28]. Higher education likely fosters greater health literacy and awareness of PA’s benefits, while higher income provides resources (time, access to facilities/equipment, potentially safer neighborhoods) enabling PA. However, our focus on leisure-time integrated PA crucially distinguishes this from studies finding higher total PA in lower-SES groups often engaged in occupational physical labor [29]. This distinction is vital; our findings imply that promoting health-enhancing leisure-time PA requires different strategies for lower-SES groups than simply acknowledging occupational activity.

Marital Status and Fertility: The finding that married individuals and those with children (especially one child) scored higher on PA requires careful interpretation. While we observed this pattern, we found that contrasting findings exist [30,31]. Potential explanations for our results include:

Shared Activities: Marriage/partnership may facilitate shared active pursuits (e.g., walking together).

Accountability and Support: Spouses might encourage or hold each other accountable for PA.

Child-Related Activity: Having children, especially young ones, inherently increases activity through play, transportation to activities, etc. Parents may also consciously model active behaviors for their children.

Life Stage: Marriage and parenthood often coincide with the peak earning/activity years (28–37 in our study), potentially confounding the effect.

We explicitly distinguish between findings supported by our data and those that are hypothesis generating. The mixed literature suggests this relationship is complex and context dependent. Factors like relationship quality, division of domestic labor, and the age/number of children likely modify the impact. Our results should not be simplistically interpreted as marriage causing higher PA, but rather that in this specific cohort, married individuals and parents reported higher levels of certain integrated PA behaviors. It is important to note that the mechanisms behind the association of marriage and fertility with PA remain speculative. This association may be partly explained by shared activities or life stage effects, though this remains speculative and warrants further investigation.

Critically, the study by Elagizi, A et al. [32] reporting a potential link between very high-intensity PA and infertility in women underscores the importance of nuance. Our fertility measure did not capture PA intensity or timing relative to childbearing attempts.

### 4.3. Specific PA Behaviors and Obesity

The granular assessment using DPABS revealed that specific behavioral skills and habits (Q5–Q8: planning, consistency, proactivity, prioritization) and achieving a minimum time commitment (Q1) were most strongly differentiated between obese and non-obese individuals. Behaviors related to mode (Q2, Q3, Q9) showed weaker or non-significant associations. This is a key contribution:

It moves beyond simply correlating total PA volume with BMI [33,34] to identify the specific types of behaviors that characterize successful weight management in daily life.

It strongly suggests that the way individuals integrate PA into their routines—specifically, their ability to plan, adapt, prioritize, and consistently achieve a baseline duration—may be more critical for weight status than the specific forms of incidental activity (like stairs or walking short trips), at least in this population. This aligns with behavioral theories emphasizing self-regulation [35].

The lack of association for Q9 (using housework/shopping as exercise) is intriguing. While conceptually appealing, it may reflect that these activities are often not performed at sufficient intensity or duration to significantly impact energy balance, or that self-perception of them as “exercise” varies greatly.

### 4.4. Study Contributions and Significance

This study offers significant contributions:

1. Contextual Focus: It uniquely targets daily life-integrated PA within the high-pressure, time-scarce environment characteristic of economically dynamic Chinese regions like the Yangtze Delta, directly addressing a major barrier to structured exercise.
2. Granular Behavioral Assessment: By employing the novel DPABS instrument, we quantified and linked specific, common, integrated PA behaviors (e.g., planning for bad weather, compensating for missed sessions, creating micro-opportunities) directly to obesity status, providing actionable insights often missing in studies using generic PA metrics or total volume.
3. Policy Relevance: Our findings directly inform the national “Healthy China 2030” strategy by identifying specific, modifiable behavioral targets (e.g., promoting planning skills via mHealth) and high-risk demographic groups for targeted public health investment.
4. Actionable Levers for Intervention: Pinpointing the specific behavioral skills (planning, consistency, prioritization) and the critical importance of achieving the ~150 min/week target (Q1) offers concrete foci for behavior change programs.

### 4.5. Explaining Disparities and Proposing Interventions

The lower PA observed in specific groups stems from complex, often intersecting barriers:

Women: time poverty due to disproportionate domestic/care burdens [36]; sociocultural norms [37]; safety concerns; potentially lower self-efficacy in certain exercise contexts. Interventions: promote time-efficient “micro-workouts” and PA integrated into domestic tasks (with realistic intensity expectations); workplace PA programs; women-only groups or safe community spaces; addressing gender norms in health messaging.

Older Adults (>58): age-related functional decline [38], comorbidities, fear of injury [39], lack of suitable programs, social isolation, potentially lower perceived benefit. Interventions: develop safe, accessible, and socially engaging community-based programs (e.g., tailored group walking, light resistance/balance training); leverage community health workers; emphasize functional benefits and fall prevention.

Lower-Income/Lower-Education Groups: limited access to facilities/safe spaces; financial constraints; time constraints due to multiple jobs/long hours; lower health literacy; potentially higher stress levels. Interventions: invest in free/low-cost community infrastructure (parks, walking paths); subsidized access to simple equipment [40]; workplace wellness initiatives; culturally tailored health education focusing on low/no-cost integrated PA; mobile health (mHealth) apps promoting “exercise snacks”.

Obese Individuals: physical discomfort/pain during activity; lower exercise self-efficacy; experiences of weight stigma in exercise settings; potential co-morbidities [41]. Interventions: focus on low-impact activities initially (swimming, cycling); emphasize non-weight-related benefits (mood, energy); provide supportive, non-stigmatizing environments; cognitive-behavioral therapy (CBT) to address barriers and build self-efficacy; telehealth coaching.

A core, evidence-based strategy emerging from our findings is promoting the development of specific behavioral skills:

Planning and Problem Solving: teaching skills to anticipate barriers (weather, schedule changes—Q5, Q6) and develop backup plans.

Habit Formation and Prioritization: encouraging strategies to embed small bouts of activity into routines and mentally prioritize PA despite busyness (Q7, Q8).

Goal Setting and Self-Monitoring: supporting individuals in setting realistic goals (starting below 150 min if needed) and tracking progress towards Q1, using tools like pedometers or apps.

Leveraging Technology: utilizing widely accessible mobile apps for reminders, micro-workout guidance, activity tracking, and social support.

### 4.6. Relevance Beyond the Chinese Context

While this study is situated within the unique socio-cultural and economic context of China, the core findings may hold relevance for other populations navigating similar modern challenges. The identified barriers—time scarcity due to high work pressure, the difficulty of adhering to structured exercise, and the critical role of self-regulatory skills for integrating PA into daily life—are not unique to China. They are increasingly common in urbanized, fast-paced societies worldwide. Therefore, the DPABS framework and the intervention strategies proposed (e.g., promoting behavioral skills via mHealth, creating supportive micro-environments) could serve as a valuable template for researchers and public health practitioners in other countries experiencing similar transitions in lifestyle and rising obesity rates.

### 4.7. Limitations and Distinguishing Evidence from Speculation

Our findings must be interpreted considering the following limitations:

Cross-Sectional Design: Precludes causal inference. While we hypothesize PA influences obesity, reverse causality (obesity limiting PA) and confounding by unmeasured factors (e.g., diet, genetics, underlying health conditions) are possible. Therefore, we state an association, not causation.

Self-Reported Data: PA behaviors, height, and weight were self-reported, susceptible to recall and social desirability bias. Although self-reported BMI has shown moderate validity in Chinese adults [42], the inability to clinically verify anthropometrics and the lack of objective PA measurement (e.g., via accelerometers) remain notable limitations. Furthermore, as a self-report instrument, DPABS is susceptible to biases such as social desirability and recall error. While we established content validity and internal consistency, the scale’s criterion validity against objective measures (e.g., accelerometers) and its generalizability to other populations require further external validation in future studies.

Sampling: Online recruitment via “Question Star” may limit generalizability, potentially underrepresenting less tech-savvy or lower-SES groups. This approach likely excluded digitally marginalized populations, such as older adults and rural residents with limited internet access, thereby skewing the socio-demographic profile of our sample and limiting the applicability of our findings to these important subgroups. Consequently, our sample may underrepresent populations with limited internet access, lower digital literacy, or those from less developed regions. This could lead to an overestimation of overall PA levels and an underestimation of the true extent of disparities, as the most vulnerable and potentially least active subgroups are less likely to be included. Our findings on socio-demographic determinants should therefore be interpreted as reflective of patterns within an online, relatively connected population, and may not fully capture the challenges faced by the digitally marginalized.

Lack of Intensity/Duration Detail: While DPABS captures frequency/habit, it does not precisely quantify intensity or exact duration of activities like walking (Q3) or housework (Q9), limiting our ability to fully assess their energy expenditure contribution. The non-significance of Q9, for example, might reflect low intensity rather than lack of value. Its criterion validity against objective PA measures requires further verification.

Crucially, we distinguish evidence-based conclusions from speculation as follows:

Evidence: We found significant associations between lower scores on specific DPABS items (Q1, Q5, Q6, Q7, Q8) and obesity. We found significant demographic differences in PA levels (gender, age, education, income). These are directly supported by our data analysis.

Plausible Explanation (Requiring Further Validation): The mechanisms linking PA to obesity (energy balance [43], insulin sensitivity [44], self-regulation) are well-established in physiology/behavioral science, though we did not measure them directly here, also, unaccounted confounders such as dietary patterns and sleep duration may influence the observed relationships. The reasons for demographic disparities (e.g., time constraints for women, resource limitations for low SES) are strongly suggested by the existing literature and our context, but specific mediating factors were not measured in depth. Intervention suggestions are logical extensions of our findings and established behavioral principles but require empirical testing in this specific population.

Speculation: The exact reasons why marriage/fertility showed positive associations in our sample, contrasting some literature, remain speculative (shared activities, accountability, child-related activity). Further qualitative or longitudinal research is needed.

## 5. Conclusions

In conclusion, this study highlights that daily life-integrated PA, particularly behaviors demonstrating planning, consistency, proactivity, prioritization, and achievement of minimum duration targets, is significantly associated with lower obesity prevalence among Chinese adults especially in the Yangtze Delta region. Significant disparities exist, with women, older adults (>58 years), individuals with lower income/education, and obese individuals engaging less in these beneficial behaviors. Addressing the obesity epidemic in China requires targeted interventions that focus on building specific behavioral skills and overcoming the unique barriers faced by these vulnerable groups, moving beyond generic PA promotion. Future research should employ longitudinal designs, incorporate objective PA measurements (e.g., accelerometers) to validate the DPABS and establish its predictive validity across diverse populations, delve deeper into the mechanisms linking specific integrated behaviors to physiological outcomes, and rigorously test the efficacy of interventions targeting the identified behavioral skills (planning, prioritization, consistency) in the identified high-risk groups within the Chinese and other relevant contexts.

### Implications for Practice and Policy

Our findings suggest that public health interventions should move beyond generic advice and instead focus on building specific behavioral capabilities. For women, time-efficient “micro-workouts” and women-only community programs can address time poverty and sociocultural barriers. For older adults (>58), safe, socially engaging activities like community-based Tai Chi, developed in partnership with clinical services, are essential. For low-SES groups, interventions should focus on improving access through subsidized home equipment and optimizing public spaces for activity. For obese individuals, initial focus should be on low-impact activities (e.g., aquatic exercise) combined with digital tools (apps) that build the self-regulatory skills (planning, consistency) highlighted by our study.

## Figures and Tables

**Table 1:** ANOVA results of the effect of each socio-demographic factor on daily physical activity (For brevity, selected demographic categories have been combined).

Group	Classification	Q1	Q2	Q3	Q4	Q5	Q6	Q7	Q8	Q9	Total
Gender	Male	3.06 ± 1.07	2.95 ± 1.16	3.31 ± 1.03	2.64 ± 1.12	2.69 ± 1.09	2.58 ± 1.02	2.71 ± 1.00	2.72 ± 1.04	3.08 ± 0.98	57.19 ± 15.51
	Female	2.70 ± 1.00	2.73 ± 1.17	3.12 ± 1.02	2.32 ± 1.04	2.53 ± 1.00	2.33 ± 0.93	2.58 ± 0.95	2.46 ± 0.95	3.02 ± 1.01	52.89 ± 14.43
	Welch F	25.106	7.001	6.872	18.897	4.649	13.18	3.751	14.008	0.61	16.966
	P	<0.001	0.008	0.009	<0.001	0.031	<0.001	0.053	<0.001	0.435	<0.001
Age	18~28	2.79 ± 1.04	2.91 ± 1.17	3.19 ± 1.03	2.50 ± 1.06	2.58 ± 0.97	2.50 ± 0.94	2.60 ± 0.93	2.54 ± 0.96	2.91 ± 0.98	54.51 ± 14.86
	28~38	3.09 ± 1.05	2.91 ± 1.17	3.41 ± 1.06	2.79 ± 1.22	2.99 ± 1.16	2.74 ± 1.11	2.95 ± 1.09	2.84 ± 1.07	3.45 ± 0.98	60.41 ± 17.31
	38~48	2.91 ± 1.05	2.69 ± 1.15	3.13 ± 1.00	2.28 ± 0.96	2.55 ± 0.99	2.25 ± 0.87	2.53 ± 0.93	2.49 ± 0.96	3.07 ± 1.01	53.12 ± 13.00
	48~58	2.80 ± 0.96	2.63 ± 1.19	3.27 ± 0.95	2.2 ± 0.98	2.44 ± 1.07	2.14 ± 0.75	2.69 ± 0.92	2.66 ± 0.93	3.25 ± 0.80	53.47 ± 10.62
	58~	2.39 ± 1.03	2.21 ± 0.96	2.54 ± 0.92	1.46 ± 0.74	1.43 ± 0.74	1.50 ± 0.75	2.00 ± 0.90	1.79 ± 0.96	2.54 ± 0.96	39.68 ± 12.27
	Welch F	3.696	4.605	5.268	16.705	21.132	17.766	7.001	7.386	11.071	14.528
	P	0.007	0.002	0.001	<0.001	<0.001	<0.001	<0.001	<0.001	<0.001	<0.001
Education background	Junior high school and below group	2.45 ± 1.01	2.49 ± 0.99	2.82 ± 1.01	1.74 ± 0.82	1.95 ± 0.94	1.89 ± 0.94	2.25 ± 0.97	2.29 ± 0.99	3.03 ± 0.90	46.45 ± 12.79
	High Schools and Junior Colleges	2.91 ± 1.13	2.76 ± 1.14	3.11 ± 0.97	2.43 ± 1.02	2.57 ± 1.08	2.35 ± 0.94	2.64 ± 0.94	2.44 ± 0.99	3.01 ± 1.10	53.81 ± 13.84
	Junior college	3.00 ± 0.96	2.71 ± 1.16	3.03 ± 0.96	2.39 ± 1.05	2.62 ± 0.95	2.49 ± 0.82	2.63 ± 0.93	2.63 ± 0.97	2.97 ± 0.95	54.38 ± 13.80
	Undergraduate	2.83 ± 1.03	2.87 ± 1.19	3.26 ± 1.05	2.51 ± 1.10	2.62 ± 1.02	2.49 ± 0.99	2.65 ± 0.97	2.57 ± 0.98	3.02 ± 1.01	55.14 ± 15.22
	Master and above group	3.15 ± 1.12	3.07 ± 1.15	3.52 ± 0.93	2.87 ± 0.97	3.12 ± 1.08	2.62 ± 0.92	2.93 ± 1.01	2.92 ± 1.09	3.43 ± 0.93	61.37 ± 15.23
	Welch F	4.569	3.136	5.735	16.608	12.139	7.328	4.224	3.369	2.838	10.427
	P	0.002	0.016	<0.001	<0.001	<0.001	<0.001	0.003	0.011	0.026	<0.001
Income	Less than 3000 yuan/month	2.69 ± 1.04	2.83 ± 1.19	3.19 ± 1.03	2.31 ± 1.09	2.45 ± 1.03	2.40 ± 1.00	2.52 ± 0.95	2.49 ± 1.02	2.94 ± 0.98	54.51 ± 14.86
	3 K~8 K/month	2.85 ± 1.01	2.83 ± 1.14	3.12 ± 1.04	2.41 ± 1.02	2.53 ± 0.96	2.38 ± 0.9	2.55 ± 0.92	2.46 ± 0.91	2.93 ± 0.97	60.41 ± 17.31
	8 k~15 k/month	2.97 ± 1.08	2.66 ± 1.16	3.25 ± 0.97	2.57 ± 1.16	2.81 ± 1.13	2.48 ± 1.00	2.82 ± 0.97	2.78 ± 1.04	3.25 ± 1.00	53.12 ± 13.00
	15,000~30,000 yuan/month	2.95 ± 0.97	3.08 ± 1.23	3.59 ± 0.87	2.89 ± 0.91	3.00 ± 1.08	2.84 ± 1.17	3.08 ± 1.19	2.84 ± 1.01	3.73 ± 0.87	53.47 ± 10.62
	Greater than 30,000 yuan/month	3.61 ± 0.94	3.3 ± 1.26	3.65 ± 1.23	3.09 ± 1.24	3.04 ± 1.15	2.74 ± 1.01	3.09 ± 1.28	3.13 ± 1.10	3.57 ± 1.08	39.68 ± 12.27
	Welch F	5.775	2.007	3.266	5.275	5.265	0.093	5.009	5.259	10.508	14.528
	P	<0.001	0.099	0.014	0.001	0.001	0.087	0.001	0.001	<0.001	<0.001
Marriage	Married	2.94 ± 1.06	2.75 ± 1.17	3.21 ± 1.04	2.43 ± 1.11	2.64 ± 1.12	2.39 ± 1.00	2.70 ± 1.05	2.63 ± 1.05	3.21 ± 1.01	55.35 ± 15.52
	Unmarried	2.76 ± 1.01	2.89 ± 1.17	3.19 ± 1.02	2.46 ± 1.06	2.55 ± 0.96	2.47 ± 0.94	2.57 ± 0.89	2.50 ± 0.93	2.89 ± 0.97	53.97 ± 14.52
	Welch F	6.628	2.776	0.128	0.193	1.548	1.541	3.858	3.733	21.481	1.817
	P	0.01	0.096	0.721	0.66	0.214	0.215	0.05	0.054	<0.001	0.178
Fertility	None	2.77 ± 1.01	2.88 ± 1.16	3.17 ± 1.02	2.47 ± 1.05	2.56 ± 0.96	2.46 ± 0.93	2.56 ± 0.89	2.50 ± 0.93	2.92 ± 0.96	53.97 ± 14.34
	1	3.00 ± 1.06	2.77 ± 1.19	3.23 ± 1.00	2.44 ± 1.08	2.69 ± 1.10	2.39 ± 0.95	2.69 ± 1.02	2.67 ± 1.02	3.18 ± 0.98	55.69 ± 14.93
	2 and above	2.83 ± 1.11	2.73 ± 1.16	3.24 ± 1.14	2.38 ± 1.21	2.50 ± 1.19	2.40 ± 1.15	2.78 ± 1.17	2.60 ± 1.16	3.26 ± 1.12	54.95 ± 17.61
	Welch F	4.465	1.229	0.361	0.336	1.604	0.55	2.911	2.704	9.008	1.186
	P	0.012	0.294	0.698	0.715	0.203	0.578	0.056	0.069	<0.001	0.307

**Table 2:** Stepwise regression analysis of factors affecting physical activity.

Factor	Unstandardized Coefficient		Standardized Coefficient	*t*	*p*	*VIF*	Confidence Interval	
	*B*	*SE*	*β*				Upper Limit	Lower Limit
(Constant)	57.145	5.908		9.672	0		45.548	68.741
Educational attainment	2.037	0.624	0.14	3.264	0.001	1.778	0.812	3.261
Personal income	1.593	0.671	0.102	2.374	0.018	1.767	0.276	2.911
Gender	−4.16	0.996	−0.136	−4.178	<0.001	1.021	−9.014	−0.871
Age group	−4.291	0.746	−0.327	−5.75	<0.001	3.118	0.753	5.729
Fertility status	3.241	1.268	0.156	2.557	0.011	3.587	−5.756	−2.826
Marital status	−4.942	2.074	−0.165	−2.383	0.017	4.587	−6.115	−2.206

**Table 3:** Results of chi-square test between obese and non-obese groups for each entry of daily physical activity.

Entry	Group	Never	Seldom	Sometimes	Frequently	Always	*χ* ^2^	*P*
Q1	Non-obese	59 (7.3%)	249 (30.9%)	293 (36.4%)	142 (17.6%)	62 (7.7%)	12.187	0.016
	Obese	11 (19%)	19 (32.8%)	20 (34.5%)	5 (8.6%)	3 (5.2%)		
Q2	Non-obese	99 (12.3%)	242 (30.1%)	238 (29.6%)	145 (18%)	81 (10.1%)	6.257	0.181
	Obese	11 (19%)	20 (34.5%)	14 (24.1%)	5 (8.6%)	8 (13.8%)		
Q3	Non-obese	34 (4.2%)	167 (20.7%)	283 (35.2%)	232 (28.8%)	89 (11.1%)	5.922	0.205
	Obese	3 (5.2%)	15 (25.9%)	26 (44.8%)	9 (15.5%)	5 (8.6%)		
Q4	Non-obese	164 (20.4%)	261a (32.4%)	258 (32%)	82 (10.2%)	40 (5%)	7.544	0.11
	Obese	20 (34.5%)	18a (31%)	14 (24.1%)	3 (5.2%)	3 (5.2%)		
Q5	Non-obese	113 (14%)	259 (32.2%)	292 (36.3%)	100 (12.4%)	41 (5.1%)	15.015	0.05
	Obese	18 (31%)	19 (32.8%)	17 (29.3%)	2 (3.4%)	2 (3.4%)		
Q6	Non-obese	127 (15.8%)	307 (38.1%)	272 (33.8%)	73 (9.1%)	26 (3.2%)	11.604	0.021
	Obese	18 (31%)	20 (34.5%)	18 (31%)	1 (1.7%)	1 (1.7%)		
Q7	Non-obese	89 (11.1%)	268 (33.3%)	308 (38.3%)	113 (14%)	27 (3.4%)	13.326	0.01
	Obese	15 (25.9%)	17 (29.3%)	21 (36.2%)	3 (5.2%)	2 (3.4%)		
Q8	Non-obese	104 (12.9%)	276 (34.3%)	295 (36.6%)	98 (12.2%)	32 (4%)	16.252	0.003
	Obese	16 (27.6%)	24 (41.4%)	16 (27.6%)	1 (1.7%)	1 (1.7%)		
Q9	Non-obese	58(7.2%)	149(18.5%)	346(43%)	197(24.5%)	55(6.8%)	2.512	0.642
	Obese	5(8.6%)	12(20.7%)	27(46.6%)	9(15.5%)	5(8.6%)		

**Table 4:** Results of *t*-test between obese and non-obese groups under each entry of daily physical activity scores.

Entry	Non-Obese	Obese	*t*	*p*
	n = 805	n = 58		
Q1	2.87 ± 1.04	2.48 ± 1.06	2.777	0.006
Q2	2.83 ± 1.16	2.64 ± 1.28	1.238	0.216
Q3	3.22 ± 1.03	2.97 ± 0.99	1.803	0.072
Q4	2.47 ± 1.08	2.16 ± 1.12	2.141	0.033
Q5	2.62 ± 1.04	2.16 ± 1.02	3.332	0.001
Q6	2.46 ± 0.97	2.09 ± 0.92	2.832	0.005
Q7	2.65 ± 0.97	2.31 ± 1.03	2.604	0.009
Q8	2.60 ± 0.99	2.09 ± 0.88	4.238	<0.001
Q9	3.05 ± 1.00	2.95 ± 1.03	0.766	0.444

## Data Availability

Due to the ethical requirements of the project, the privacy of the respondents must be protected, and the data cannot be made public for the time being.
